# Peroral Endoscopic myotomy (POEM) in pediatric achalasia: a retrospective cohort on institutional experience and quality of life

**DOI:** 10.1186/s13023-025-03565-y

**Published:** 2025-01-25

**Authors:** Thijs Kuipers, Carlijn Mussies, Aaltje Lei, Gwen M.C. Masclee, Marc A. Benninga, Paul Fockens, Barbara A.J. Bastiaansen, Albert J. Bredenoord, Michiel P. van Wijk

**Affiliations:** 1 Department of Gastroenterology and Hepatology, Amsterdam University Medical Centers, University of Amsterdam, Amsterdam, Netherlands; 2 Amsterdam Gastroenterology, Endocrinology and Metabolism, Amsterdam, Netherlands; 3 Department of Pediatric Gastroenterology and Nutrition, Amsterdam UMC, Emma Children’s Hospital, University of Amsterdam, Amsterdam, 1105 AZ the Netherlands; 4 Department of Pediatric Gastroenterology and Nutrition, Amsterdam UMC, Emma Children’s Hospital, Vrije Universiteit, Amsterdam, The Netherlands

**Keywords:** Esophagus, Motility disorder, Achalasia, Endoscopy, Quality of life

## Abstract

**Background:**

Achalasia is a rare esophageal motility disorder with an estimated annual incidence of 1–5/100.000 and a mean age at diagnosis > 50 years of age. Only a fraction of the patients has an onset during childhood (estimated incidence of 0.1–0.18/ 100.000 children per year). No curative treatment is currently available. Peroral Endoscopic Myotomy (POEM) is a widely accepted treatment option to improve symptoms in adults. Studies evaluating safety and efficacy of POEM in children are scarce and no data exist regarding the quality of life in patients after POEM.

**Methods:**

We evaluated the effectiveness and safety of POEM in a cohort of children that was treated for achalasia and we prospectively evaluated their quality of life. We compared the results to a previous cohort evaluating Pneumatic Dilation (PD) and Laparoscopic Heller’s Myotomy (LHM) in children with achalasia.

**Results:**

Thirty-three achalasia patients (age at time of POEM 14.1(± 2.5) years, 54.5% female) were included. Twenty-nine (87.8%) percent had received previous treatment (PD (*n* = 20); LHM (*n* = 1); PD + LHM (*n* = 7); PD + Botox (*n* = 1). POEM was technically successful in all patients and no major complications occurred. Mean follow-up duration was 33 (± 25) months. Twenty three (70%) patients did not need retreatment after POEM during the follow up period. Quality of life after POEM did not differ from the population norms. Patients with an Eckardt score > 3 had a significantly worse general (Kidscreen-52: physical score 44.7 vs. 52.4; *p* = 0.011; mental score: 42.5 vs. 51.3; *p* = 0.038) and disease specific (35 vs. 16; *p* = 0.017) quality of life compared to those with an Eckardt ≤ 3. The SF-36 mental health component score was significantly lower (44.2 vs. 53.1; *p* = 0.036) in patients treated with POEM compared to those treated with PD and LHM. These lower scores could be related to a selection bias, as more severe patients received POEM, and other influences such as the Corona pandemic. However, the overall, quality of life after POEM was not significantly different to PD and LHM.

**Conclusion:**

POEM is an effective and safe treatment for achalasia in children. Quality of life after POEM is comparable to the results obtained after PD and Heller.

**Supplementary Information:**

The online version contains supplementary material available at 10.1186/s13023-025-03565-y.

## Introduction

Achalasia is a rare esophageal motility disorder with an estimated incidence of 0.1–0.18 per 100.000 children per year [1, 2]. The disease is characterized by altered or absent peristalsis of the esophageal body and incomplete relaxation of the lower esophageal sphincter (LES), leading to symptoms such as dysphagia, regurgitation, retrosternal pain and weight loss [3]. A delay in diagnosis often occurs because of the rarity of the disease resulting in a delayed recognition due to attribution of symptoms to gastroesophageal reflux disease, eosinophilic esophagitis or eating disorders.

Currently there is no curative treatment for achalasia and treatment options are aimed at symptom relieve by reducing LES pressure. In pediatric achalasia the most frequently applied treatment options are pneumatic dilation (PD) and laparoscopic Heller’s myotomy (LHM). No clear consensus is available on the optimal choice of treatment [4]. Pneumatic dilation is an effective non-surgical treatment. However, as relapses occur frequently (treatment failure 34–90%) most patients require multiple redilations [1, 5]. Laparoscopic myotomy on the other hand, is seen as a more permanent treatment option with a better long-term outcome of 85% in children, but is associated with a higher complication rate [6]. 

Peroral endoscopic myotomy (POEM) is an endoscopic technique first performed in 2008 in adults [7]. POEM allows endoscopic myotomy of the LES and esophageal body. In adults, it provides a more permanent treatment and relieve of symptoms than PD, without abdominal incision and post-operative pain which occurs in LHM [6]. As a full thickness myotomy is not necessary to obtain reduction in LES pressure and clinical success, a further advantage of POEM may be the selective dissection of the circular muscles which leaves the longitudinal layer intact [8]. A large randomized controlled trial showed a two year efficacy rate of 92% vs. 54% for POEM and PD, respectively [9]. At five year follow-up, an efficacy of 81% vs. 40% (POEM vs. PD, respectively) remained [10]. However, reflux esophagitis was seen significantly more often in patients treated with POEM [9, 10]. Although studies evaluating safety and efficacy of POEM in pediatric patients are scarce and small, single center and uncontrolled, the results are promising with a reported treatment success of achalasia symptoms in 94% of patients [11]. 

Previous research has shown that quality of life in pediatric achalasia patients is lower in comparison to the healthy population and even lower than children with inflammatory bowel disease [12]. We know that quality of life improves after successful achalasia treatment [1]. However, it is unknown if quality of life is comparable after POEM as opposed to PD or LHM.

The aim of this study is therefore to evaluate effectiveness and safety of POEM in children with achalasia and compare quality of life of patients treated with POEM to those who had PD or LHM.

## Methods

We reviewed the medical records of all achalasia patients who received POEM at the Amsterdam University Medical Centers (AUMC) before the age of 18 years, and before April 2022 with a minimum follow-up time of 6 months. In the Netherlands, achalasia care, including POEM treatment, is centralized to our center, therefore, we covered the entire Dutch pediatric achalasia population. The study was waived of full ethical review by the Amsterdam UMC medical ethical review board based on the nature of the study (W13_291#13170360).

Patient demographics and medical history were collected from the medical records. The following items were included: patient characteristics (i.e. age at diagnosis, gender and medical history), type of achalasia, diagnostic investigations (including high resolution manometry (HRM) and barium esophagram) and previous achalasia treatments. All patients were offered a standardized follow-up, as per routine patient care. This includes routine follow-up visits, an endoscopy and 24 h pH impedance measurement 3 months after POEM and routine oral PPI.

Follow-up visits at 6 months, 1 year and 2 years (if available) post POEM were taken into account, even if these visits took place after the patient reached the age of 18 years. Treatment success was evaluated based on the need for retreatment. Clinical symptoms, Eckardt score, HRM and/or barium esophagram (if performed) were also evaluated. Presence of reflux disease was assessed based on symptoms, gastroscopy and/or 24 h pH-impedance measurement and use of PPI medication. Procedure related complications were evaluated and classified according to the criteria found in supplement 1.

All patients were contacted by telephone by a research nurse. Patients, or their parents if age not appropriate, were asked about their current achalasia (dysphagia, odynophagia, regurgitation, retrosternal pain and weight loss– by the means of the Eckardt questionnaire) and reflux symptoms (acid reflux, chest pain, cough– by the means of the Reflux Disease Questionnaire) including PPI use. The Eckardt score (positive if > 3) [13] and GERDQ questionnaire (cut-off 8) [14] were completed during the telephone interview. Quality of life was assessed using a general quality of life and disease specific quality of life questionnaire by means of an online survey. If a patient was aged ≥ 18 years at the time of the phone interview we used the 36-Item Short Form Health survey (36-SF) [15] and the Health related QoL questionnaire [16]. Patients aged < 18 years filled out the Kidscreen-52 [17] and disease specific QoL [18]. The results from the quality of life questionnaire were compared to the population norms when applicable. Furthermore, we compared our results to a previous study evaluating the pediatric achalasia population in our center. This study consisted of 87 patients diagnosed with achalasia < 18 years old between 1990 and 2013 and assessed treatment success of the then available treatment options; PD and LHM, as well as symptoms and quality of life at an additional follow-up [1]. 

### Statistical analysis

Descriptive statistics were presented as percentage for categorical data and as mean with standard deviation (SD) or median with interquartile range (IQR) for continuous variables. Analysis of categorical data was performed using Chi-Square or Fisher exact tests. Continuous data were compared using the unpaired Student t-test for normally distributed data and the Mann-Whitney U test for non-normally distributed data. A p-value of < 0.05 was considered significant. SPSS statistics (version 28; SPSS, Chicago, Illinois, USA) was used for statistical analysis.

## Results

We identified 34 patients with achalasia (mean age at time of POEM 14.1 (2.5) years, 18 female (54.5%)) that underwent POEM before the age of 18 years at the AUMC between October 2015 and April 2022 (Table 1). One patient was later diagnosed with pseudo-achalasia based on a malignancy, this patient is excluded from the analysis. The mean age at achalasia diagnosis was 12.09 (2.69) years. Patients reported a mean Eckardt score of 6.29 (2.05) at time of diagnosis, all Eckardt scores were ≥ 3. HRM data was available in 32 (97.0%) of 33 patients at time of diagnosis and showed a median integrated relaxation pressure (IRP-4) of 36.4 (26.0-50.4) mmHg (normal < 15mmHg). Based on HRM and according to the Chicago classification of Esophageal Motility Disorders (version 3.0) the following achalasia subtypes were seen; 12 (36.4%) patients had subtype I, 19 (57.6%) patients had subtype II, one (3.0%) patient had subtype III achalasia and in one (3.0%) patient the type of achalasia was unknown. Twenty-nine of 33 patients (87.8%) had received a different treatment prior to POEM. This was PD (20 [60.6%] of 33), LHM (1 [3.0%] of 33) PD + LHM (7 [21.2%] of 33) or PD + Botox (1 [3.0%] of 33). The time between diagnosis and POEM was 12 (6–26) months.

**Table 1:** Baseline characteristics of included patients (*n* = 33)

	*n*	%	Mean (SD)/ Median (IQR range)
*Demographics*			
Sex			
Male	15	45.5	
Female	18	54.5	
Age at diagnosis (years)			12.09 (3.69)
Type of achalasia			
Achalasia type I	12	36.4	
Achalasia type II	19	57.6	
Achalasia type III	1	3.0	
Unknown	1	3.0	
BMI (kg/m2)			17.99 (4.38)
Eckardt score at diagnosis			
Total score			6.29 (2.05)
Dysphagia			2.39 (0.74)
Retrosternal pain			1.04 (0.96)
Regurgitation			1.48 (1.12)
Weight loss			1.45 (1.02)
Stasis on barium esophagram (T = 5 min) at diagnosis			
Yes	22	66.7	
No	3	9.1	
Unknown	1	3.0	
Not performed	7	21.2	
HRM			
HRM performed?	32	97.0	
Baseline pressure			50.7 (39.7–65.1)
IRP-4			36.4 (26.0-50.4)
Previous treatment	29	87.8	
PD	20	60.6	
LHM	1	3.0	
PD + LHM	7	33.0	
PD + botox	1	3.0	
SD: standard deviation; IQR: inter quartal range; BMI, body mass index; n, number of patients; HRM, high resolution manometry; IRP-4: integrated relaxation pressure; PD: pneumatic dilation; LHM, laparoscopic Heller’s myotomy			

### POEM procedure

The POEM procedure was technically successful in all patients. In 27 [81.8%] of 33 patients a myotomy of the anterior side was done and 6 [18.2%] patients received a posterior myotomy. The mean length of the myotomy was 9.38 (2.08) cm and the median length of myotomy extended in the stomach was 3 [2, 3, 4] cm. The median duration time of the procedure was 52 (42–61) minutes. The median hospital stay was one (1–1) night.

### Safety

No major complications occurred during the POEM procedure or in the thirty days thereafter. Four intra-procedural events occurred. Three (9.1%) patients had minor intra-procedural bleedings that could be resolved with coagulation during the procedure. In one (3.0%) patient a small mucosal tear occurred that could be closed with one clip during the procedure.

### Treatment success

**Fig. 1:**
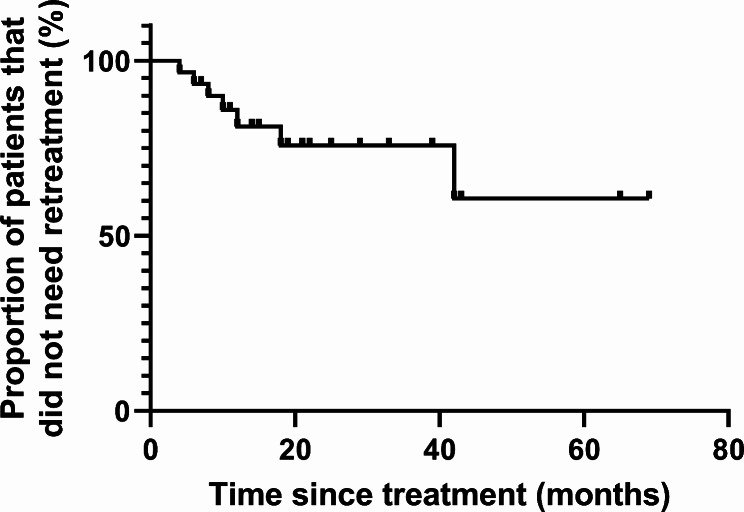
Log-rank survival curves for the proportion of patients that did not need retreatment

The mean follow-up duration of all patients was 33 (25) months. During follow-up 23 [69.7%] of 33 patients did not need retreatment and 3 (9.1%) patients were lost to follow-up. A log-rank survival curve for the proportion of patients that did not need retreatment is shown in Fig. 1. Of the seven patients that needed retreatment, four (57.1%) were treated with PD only and received the first dilation 6, 8, 10 and 12 months after POEM. One [14.3%] patient received PD 3 months and LHM 13 months after POEM. One [14.3%] patient received a re-POEM 42 months after the initial POEM. And one [14.3%] patient received a re-POEM at 18 months followed by PD 29 months after the first POEM.

### Regular follow-up

At 3 months after POEM a follow-up visit was completed in 28 (84.8%) of 33 patients. 27 patients (96.4%) did not need additional treatment. Five (17.9%) of these patients reported an Eckardt score > 3. One patient was retreated with PD within 3 months after treatment with POEM. Endoscopy was performed in 10 (30.3%) of 33 patients off PPI and showed LA grade A esophagitis in one (10.0%) patient and LA grade B esophagitis in 2 (20%) patients.

Follow-up data at 1-year after treatment with POEM was available in 21 (77.8%) of 27 patients. In the other six of the 33 patients included in the trial POEM was performed less than one year ago. 18 (85.7%) of these 21 patients did not need additional treatment after POEM. Five (23.8%) patients reported an Eckardt score > 3, three of these of them patients received treatment with PD between 3 months and 1 year after POEM, one of these patients needed a second dilation series.

### Additional follow-up

In total 29 (87.9%) of 33 patients were included for additional follow-up questionnaires and interview. Despite multiple attempts, the other 4 patients could not be reached. At time of additional follow-up, patients had a mean current age of 16.66 (2.79) years at time of follow-up. The mean Eckardt score was 2.43 (1.75); 13 (44.8%) of 29 patients reported an Eckardt score > 3. An overview of the Eckardt subscores can be found in Table 2. At the time of additional follow-up 6 (18.2%) patients reported a GERDQ of 8 or higher, 12 (26.4%) patients were using PPI daily. Two (6.9%) of 29 indicated coughing.

**Table 2:** Follow-up questionnaires of included patients (*n* = 29)

	*N* (%)		Mean (± SD)/ Median (IQR range)
Current age			16.66 (2.79)
Eckardt score			
Total score			2.43 (1.75)
Dysphagia			1.17 (0.97)
Retrosternal pain			0.66 (0.61)
Regurgitation			0.45 (0.69)
Weight loss			0.07 (0.26)
GERDQ 8 or higher?			
Yes	6 (18.26%)		
No	21 (81.8%)		
PPI use			
Yes	12 (36.4%)		
No	16 (48.5%)		
Follow-up duration (months)			33.21 (25.41)
Re-treatment?			
Yes	7 (21.2%)		
No	23 (69.7%)		
Unknown	3 (9.1%)		
Type re-treatment			
PD	4 (57.1%)		
POEM	1 (14.3%)		
PD + POEM	1 (14.3%)		
PD + LHM	1 (14.3%)		
Time between POEM and retreatment (months)			10 (6–18)
N, number of patients; SD: standard deviation; IQR: inter quartal range; PPI: proton pump inhibitor; PD: pneumatic dilation; POEM: peroral endoscopic myotomy; LHM, laparoscopic Heller’s myotomy			

### Quality of life

Fourteen (77.8%) out of 18 patients younger than 18 years of age completed the Kidscreen-52 and disease specific QoL (DSQoL). On the Kidscreen-52 our cohort did not score significantly different on any domain compared to the population norms, except for the ‘bullying’ domain where achalasia treated patients scored better (i.e. were bullied less often, *p* = 0.013). An overview of the scores on the different subdomains can be found in supplement 1. A total median pediatric achalasia-specific DSQoL score of 21 (14.8–26.3) was reported. The subdomains swallowing problems, friends and family and feelings scored 8 (4.8–16), 5.5 (1.8–11.3) and 7.5 (1.8–12) respectively.

Six (54.5%) of the 11 patients older than 18 years old at time of the additional follow-up completed the achalasia-specific HRQoL with a median score of 17 (12.8–22.5). The SF-36 showed a mental component score of 44.2 (35.8–51.0) and physical component score of 51.9 (41.2–58.3).

### Post-hoc analysis

We performed a post-hoc analysis where we created multiple subgroups. First the cohort was divided based on Eckardt score; one group had an Eckardt score ≤ 3 and the other group an Eckardt score of > 3 at the additional follow-up.

In the patients who were younger than 18 years of age at the time of the additional follow-up and had an Eckardt score > 3, we found a significantly lower score on the Kidscreen-52 physical well-being subscore (44.7 (32.7–47.1) vs. 52.4 (47.1–59.4) *p* = 0.011) and moods and emotions subscore (42.5 (37.8–49.1) vs. 51.3 (47.2–54.0) *p* = 0.038) compared to the group of children with an Eckardt score ≤ 3, Patients younger than 18 years old with an Eckardt score above three also scored significantly higher on the total achalasia-specific DSQoL score (indicating more symptoms) (35 (17–46) vs. 16 [8, 9, 10, 11, 12, 13, 14, 15, 16, 17, 18, 19, 20, 21] *p* = 0.017) and on the friends and family subdomain (11 [7, 8, 9, 10, 11, 12, 13, 14, 15, 16] vs. 3 [1, 2, 3, 4, 5, 6] *p* = 0.017) compared to patients with an Eckardt score below three.

In patients older than 18 years of age we did not find a significant difference on the SF-36 mental component score (47.7 (40.7–51.6) vs. 38.4 (28.0-50.9), *p* = 1.000) and physical component score (53.0 (41.4–58.5) vs. 50.8 (40.8–58.2), *p* = 0.700) or the achalasia-specific HRQoL score (22 (18–24) vs. 13 [12, 13, 14, 15, 16], *p* = 0.100) comparing patients with an Eckardt score of > 3 and patients with an Eckardt score below three.

Additionally, we performed a post hoc analysis where the cohort was divided in a subgroup of patients that were retreated after POEM and patients that did not receive retreatment. This did not result in a significant difference in quality of life on all questionnaires between both subgroups. Furthermore, we divided the cohort is a subgroup who were primarily treated with POEM and patients who had POEM after PD and/or LHM. We did not find a difference in quality of life.

### Comparison to previous cohort of patients treated by PD or LHM [1]

We compared our results to a previous study evaluating the pediatric achalasia population in our center. This study consisted of 87 patients (mean age 11.4 (3.4), 60% male) diagnosed with achalasia < 18 years old between 1990 and 2013. Initial treatment was PD in 79% and LHM in 21% patients. Retreatment was necessary more often after PD compared to LHM (88% vs. 22%). A full overview of the demographics can be found in the original publication [1]. The percentage of patients that participated in the additional follow-up (83% vs. 78%) and the number of patients with an Eckardt score of > 3 (44.5% vs. 45%) was comparable in the previous cohort vs. this study.

**Fig. 2:**
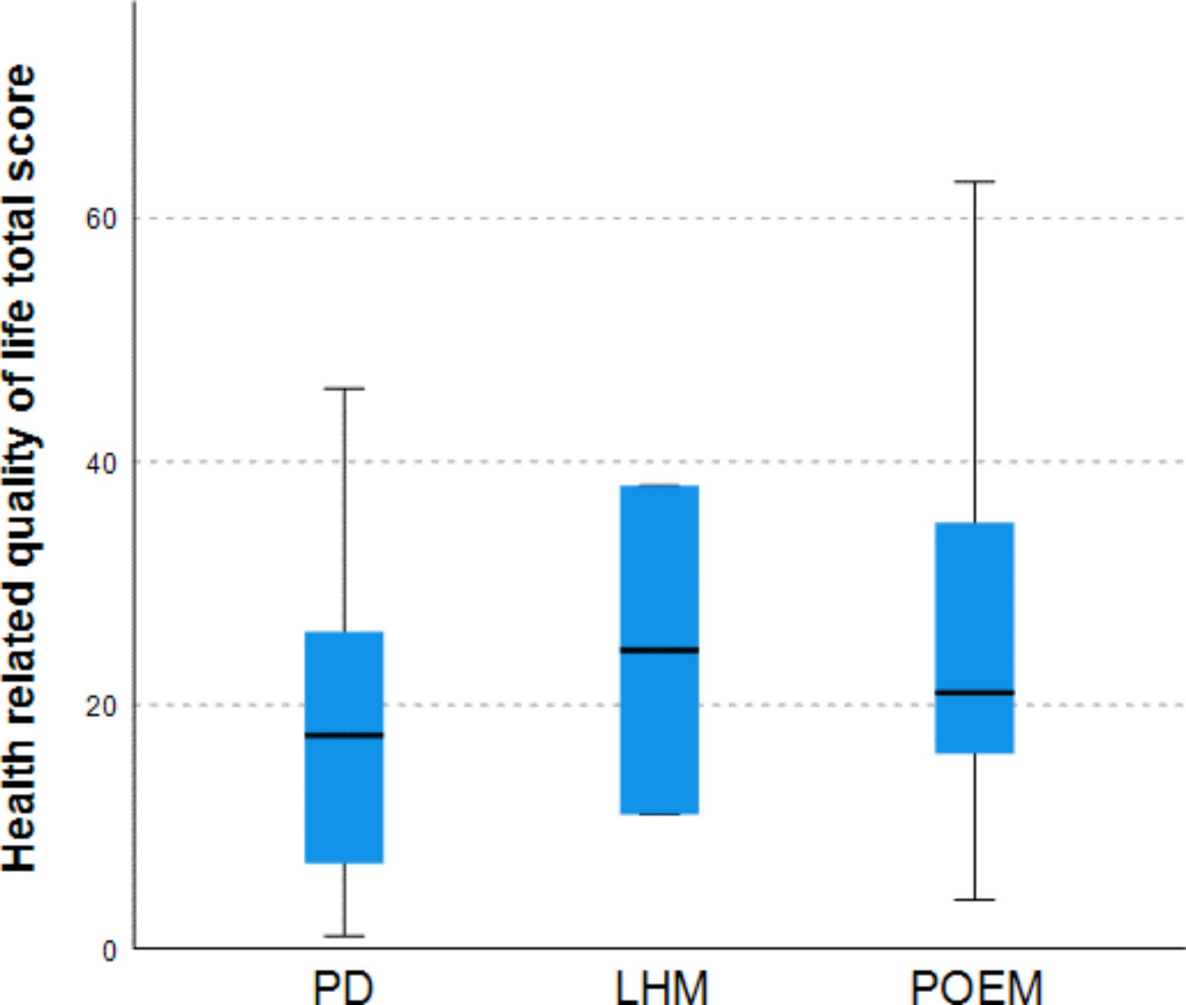
Health related quality of life after PD, LHM and POEM in children

Patients younger than 18 years at the time of the additional follow-up completed the Kidscreen and achalasia-specific DSQoL questionnaires. When comparing the Kidscreen domains in POEM treated patients with patients treated by PD or LHM, we noticed that POEM treated patients scored significantly worse on ‘self-perception’ (44.6 (40.2–53.0) vs. 53.2 (48.7–60.5) *p* = 0.036) and ‘school’ (49.6 (44.6–54.8) vs. 56.4 (50.8–61.9), *p* = 0.018). The total pediatric achalasia-specific DSQoL score did not significantly differ between the two cohorts (21 (14.8–36.3) vs. 17.5 (8–29), *p* = 0.341). Figure 2.

**Fig. 3:**
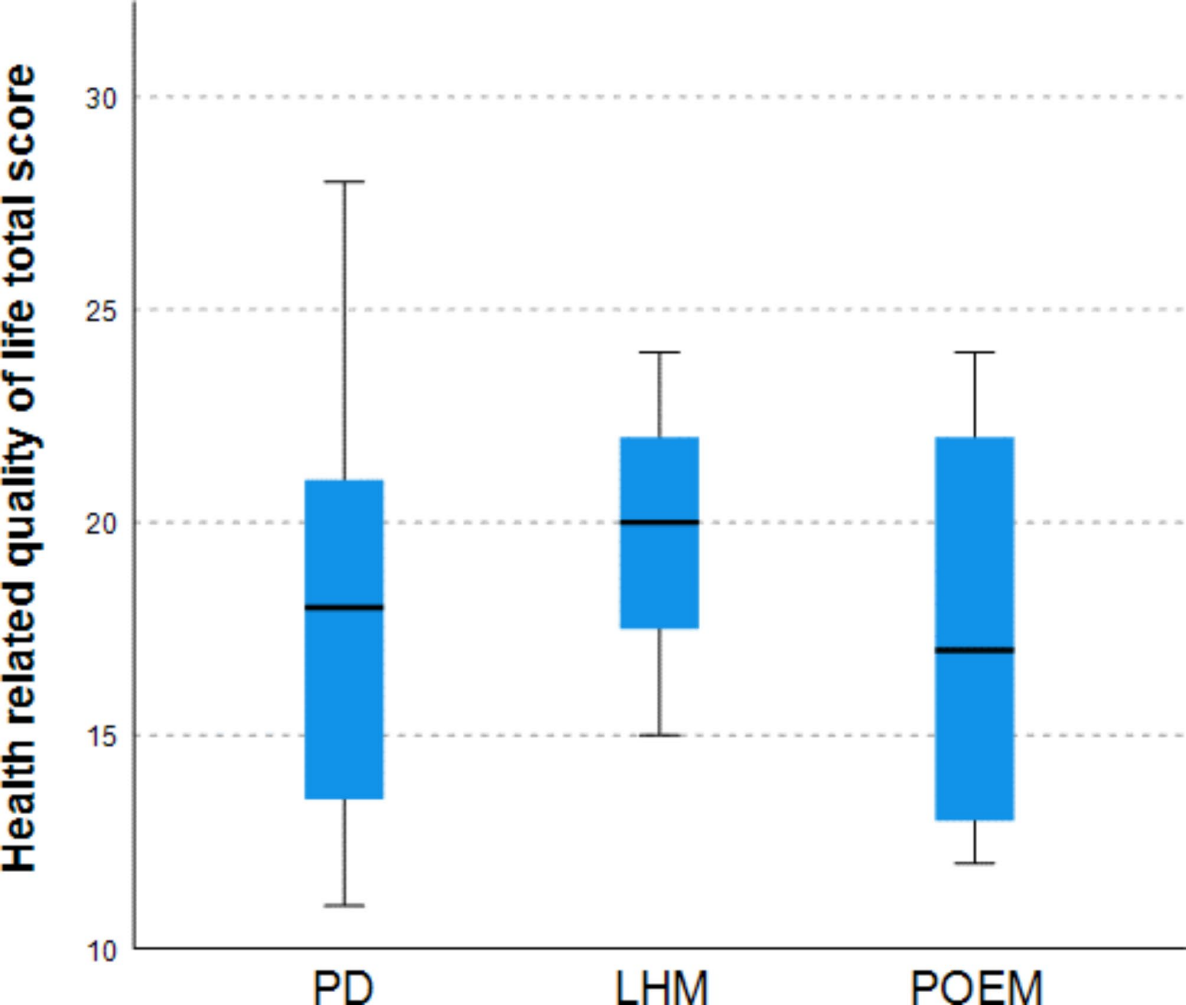
Health related quality of life after PD, LHM and POEM in adults

Patients of 18 years and older at the time of the additional follow-up completed the SF-36 and achalasia-specific HRQoL questionnaires. When comparing the scores of these questionnaires to the previous cohort of patients treated with PD or LHM we did not see a significant difference in achalasia-specific HRQoL (17 (12.8–22.5) vs. 19 [15, 16, 17, 18, 19, 20, 21, 22], *p* = 0.713) and SF-36 physical component score (51.9 (41.2–58.3) vs. 53 (49.1–57.0), *p* = 0.849) between the current and the previous cohort (Fig. 3). However the SF-36 mental component score (44.2 (35.8–51.0) vs. 53.1 (47.7–57.5), *p* = 0.036) was significantly lower in patients treated with POEM compared to patients treated with PD and LHM.

## Discussion

This study confirms that POEM is safe and effective for treatment of symptoms in children with achalasia. Furthermore, quality of life is comparable to the general population and achalasia patients treated with PD or LHM. This suggests that POEM should be considered as an effective treatment option in children with achalasia, both for relieve of symptoms and to improve overall wellbeing as well.

In this retrospective cohort 70% of the patients did not require retreatment after POEM during the course of the considerable follow-up (mean 33 months). A recent meta-analysis evaluating POEM in children with achalasia showed a success rate of 89% in pooled studies with > 2 year follow-up [19]. This difference in clinical success-rate from the meta-analysis and our study may be explained because the number of treatment naive patients was higher in the studies included in the meta-analysis, while POEM was reserved in our series for the patients unresponsive to other treatments. Until recently, we only considered POEM a suitable treatment option in children when PD and/or LHM failed. Only four (12%) patients were primarily treated with POEM in our cohort, the percentage of treatment naive patients in the studies included in the meta-analysis ranged from 50 to 85% [19]. The lower treatment success rate may thus explained by the selected population.

We found that 21% of patients reported a GERDQ score of 8 or higher at the additional follow-up, which is highly suggestive for reflux disease. This percentage is somewhat higher compared to the presence of reflux symptoms in children after POEM in other studies; 13.7% (95% CI 10.2–18.1%) [19]. However, this concerns a pooled percentage of a few retrospective and one prospective study with great heterogeneity on the definition of reflux disease. These factors could well have led to a selection and reporting bias. Furthermore, none of the studies included in the review evaluated GERD using the GERDQ questionnaire. The occurrence of reflux symptoms after POEM in adults seems to be much higher (41%) compared to our numbers (22%) [10]. The rate of erosive esophagitis was 30% 3 months after POEM in our cohort compared to 26.3% (95% CI 17.5–37.7%) in children after POEM in previously published papers [19]. Importantly, only 1 out of 3 patients underwent endoscopy 3 months after POEM. Additionally, we know from adult achalasia patients that erosive esophagitis may be present irrespective of presence of symptoms, therefore the presence of reflux esophagitis may be underestimated. However, as endoscopy is offered routinely to all patients, we would expect patients with symptoms to opt for endoscopy more often. Which may lead to overestimation of reflux esophagitis. We are not able to compare other objective criteria for reflux such as the results of 24-pH impedance measurements in our cohort to previously reported numbers, since only one patient at 3-months follow-up and one patient at 1-year follow-up underwent pH-impedance measurements.

At time of the additional follow-up, 45% of patients reported an Eckardt score > 3, suggestive of recurrent disease. This number is comparable to the percentage found in our previous cohort of achalasia patients treated with PD of LHM; 44.5% of the patients reported an Eckardt score > 3 at the additional follow-up [1]. Although widely used, the Eckardt score is not an ideal measure in children. A study identified the evaluation of weight loss and chest pain to decrease the reliability and validity of the score [20]. Since both cohort studies found a relatively high Eckardt score in patients that did not actively seek medical attention it can be argued that the Eckardt score is not well validated in children with achalasia. It might be necessary to use a different cut-off of the Eckardt score in children compared to adults or additional questions may need to be added. Therefore, the cut-off should be used with precaution when evaluating symptoms or treatment failure in children with achalasia.

We found POEM to be a safe procedure with technical success in all patients and only minor complications in three. All of these complications could be treated during the procedure and no further treatment or prolonged admission was required. This is even lower compared to a meta-analysis evaluating POEM in children were minor complications were reported in one-fourth of children [19]. 

No significant differences in achalasia specific quality of life between the patients treated with POEM in this study and those treated with LHM/ PD in the previous cohort were found based on the DSQoL and HRQoL questionnaire. Patients that completed the SF36 questionnaire at the moment of additional follow-up reported a lower mental component score; an indication of poorer mental health, compared to the previous cohort [1]. This might be explained by the fact that 88% of the patients in this cohort already received another treatment prior to POEM. These patients were treated with PD or Heller previously and now underwent POEM due to recurrence of symptoms. In the previous cohort, the patients that needed multiple treatments was lower compared to our current cohort(75% vs. 88% respectively) [1]. The larger number of patients with recurrence of symptoms and the need for retreatment which may have impacted mental health in a negative way. Furthermore, our current cohort of patients treated with POEM filled out the questionnaires after the Covid-19 pandemic, whereas the cohort treated with PD/Heller did this prior to the pandemic. It is known that the pandemic had significant effect on the mental health of adolescents [21]. This might also be an explanation for the poorer mental health score in our current cohort.

We found no significant difference in patients treated primarily with POEM compared to the PD/LHM cohort. This lack in significance might be a result of the very low number of patients primarily treated with POEM. As POEM is a relatively new treatment, it was previously seen as a rescue treatment. Further research is necessary to evaluate quality of life after POEM. A pediatric study comparing PD and POEM as first line treatment has started [22]. 

There are some limitations of this study that need to be acknowledged. First, the retrospective nature of the study could result in selection biases. We minimized this bias by reaching the majority (88%) of patients by telephone and therefore being able to prospectively evaluate quality of life after POEM.

Second, we used the Eckardt score to evaluate disease activity, which is not an ideal measure in children as mentioned previously. However, this is the only objective measure that is widely used in clinical practice to assess achalasia symptoms. Choices on treatment were not based on Eckardt scores only but included additional observations (such as additional history, HRM and/or barium esophagram).

The strengths of this study are that we were able to include a large prospective cohort of pediatric achalasia patients that were treated with POEM,. Furthermore, we were able to compare quality of life in this cohort to our previous cohort of achalasia patients treated with PD or LHM [1]. 

## Conclusions

We showed that POEM is an effective and safe treatment for achalasia in a cohort of children that had previously failed PD and/ or LHM POEM. POEM in these children resulted in a good quality of life in pediatric achalasia compared to the population norms and was largely comparable to the results obtained after pneumatic dilation and Laparoscopic Heller myotomy.

## Electronic supplementary material

Below is the link to the electronic supplementary material.

Supplementary Material 1

## Data Availability

The datasets used and/or analyzed during the current study are available from the corresponding author on reasonable request. Data cannot be openly shared due to participant privacy.

## Abbreviations

PD: Pneumatic dilation
POEM: Peroral endoscopic myotomy
LHM: Laparoscopic heller’s myotomy
LES: Lower esophageal sphincter
HRM: High resolution manometry
