# Supplementary material for: Peroral Endoscopic myotomy (POEM) in pediatric achalasia: a retrospective cohort on institutional experience and quality of life
Source: Orphanet J Rare Dis. 2025 Jan 25;20:39. doi: 10.1186/s13023-025-03565-y (PMC11765891; doi:10.1186/s13023-025-03565-y)
Supplement: Supplementary file 1 — Supplementary Material 1 [file 13023_2025_3565_MOESM1_ESM.docx]

**Supplemental material**

Classification complications

Definition of major and minor complications

Complications were defined as any unwanted event that occurred during treatment or secondary to the treatment during follow-up.

Complications were defined as major based on the following criteria

- Unexpected hospital admission for >24 hours or prolongation of a planned hospital admission for >24 hours related the treatment

- Admission to a medium or intensive care related to the treatment

- Additional endoscopic procedures within 24 hours after the treatment

- Need of blood transfusion after the treatment

- Death, related to the treatment

Complications not fulfilling the above described criteria were classified as minor.

**Supplement Table 1.** Kidscreen-52 subscores (n=14)

|  | Median (IQR range) |
| --- | --- |
| Physical well-being | 47.1 (43.7-54.2) |
| Psychological well-being | 50.6 (44.6-55.3) |
| Moods and emotions | 47.2 (42.5-51.3) |
| Self-perception | 44.6 (40.2-53.0) |
| Autonomy | 49.7 (44.8-57.3) |
| Parent relation and home life | 54.6 (47.1-60.4) |
| Peers and social support | 48.4 (42.9-53.0) |
| School environment | 49.6 (44.6-54.8) |
| Social acceptance (bullying) | 48.1 (42.2-48.1) |
| Financial resources | 59.6 (53.9-62.9) |
